# Supplementary material for: Surface-binding molecular multipods strengthen the halide perovskite lattice and boost luminescence
Source: Nat Commun. 2024 Jul 24;15:6245. doi: 10.1038/s41467-024-49751-7 (PMC11269598; doi:10.1038/s41467-024-49751-7)
Supplement: Supplementary file 1 — Supplementary Information [file 41467_2024_49751_MOESM1_ESM.pdf]

Supplementary Information for:

Surface-Binding Molecular Multipods Strengthen the Halide Perovskite Lattice and Boost Luminescence

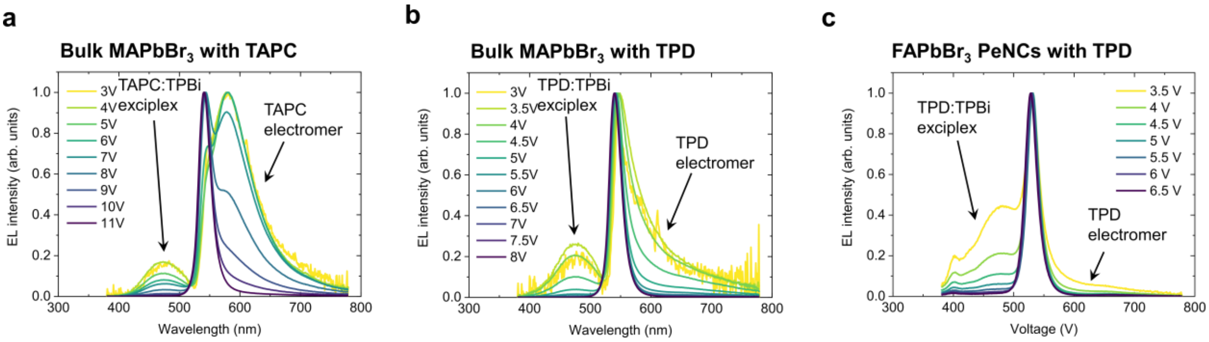

Supplementary Fig. 1 | EL spectrum of PeLEDs with hole transporting-CMMs. a, bulk $MAPbBr_3$ with TAPC. **b**, bulk $MAPbBr_3$ with TPD. **c**, $FAPbBr_3$ PeNCs with TPD.

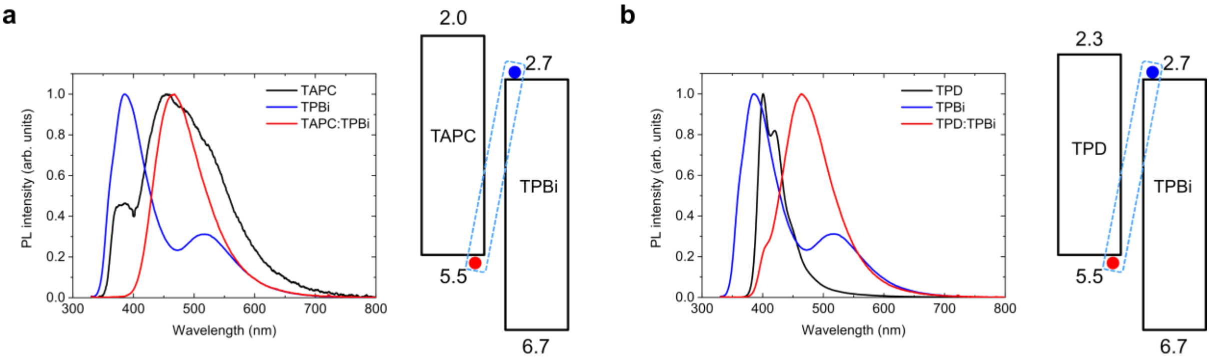

Supplementary Fig. 2 | PL spectrum of hole transporting-CMM-TPBi mixture films. a, TAPC, TPBi, and a mixture of TAPC and TPBi. **b**, TPD, TPBi, and mixture of TPD and TPBi.

CMMs with electron transporting moieties were studied in the main text. We also tested CMMs with hole transporting materials such as TAPC (di-[4-(N,N-ditolyl-amino)-phenyl]cyclohexane) and TPD (N,N′-bis(3-methyl-phenyl)-N,N′-diphenylbenzidine) using the same fabrication method shown in the main text. All devices incorporating TAPC and TPD showed exciplex emission and parasitic emission (Supplementary Fig. 1). We measured the photoluminescence spectra of TAPC and TPD mixed with TPBi and found that they form exciplex, leading to the broad emission around 470 nm. Since the EML is in contact with TPBi ETL in the device, mixing hole transporting CMMs always leads to the interfacial exciplex formation with TPBi[1]. On the other hand, since insulating PFI in the GraHIL prevents the direct contact between PEDOT:PSS hole injection layer and CMMs incorporated in the EML, electron transporting materials can be used as CMM without the formation of interfacial exciplex. The EL emission with lower energy than that of the perovskite can be attributed to the electromer emission of TAPC and TPD, respectively[2,3].

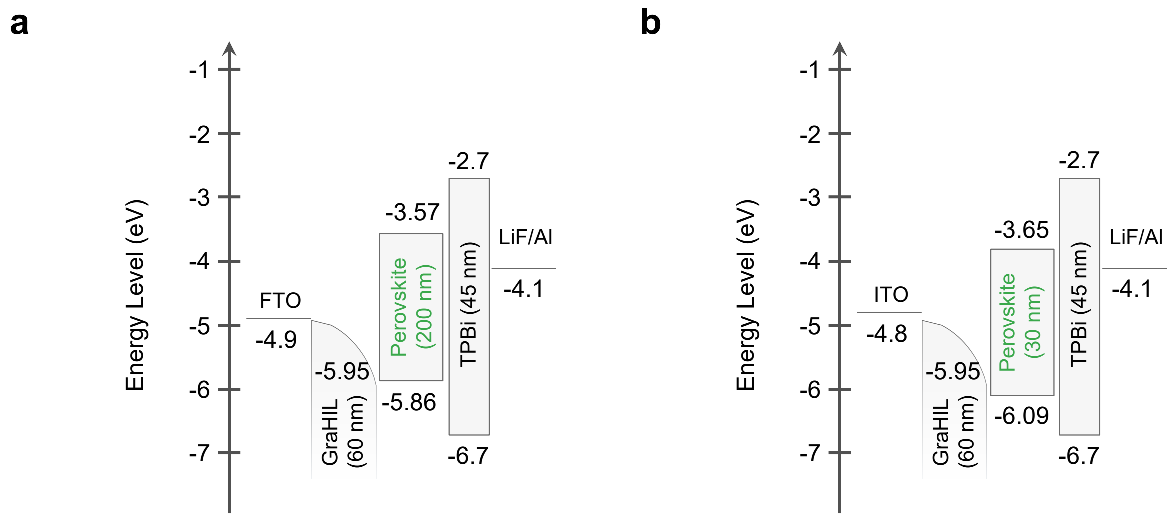

Supplementary Fig. 3 | Device structure of PeLEDs. a, Bulk perovskite, **b**, PeNC

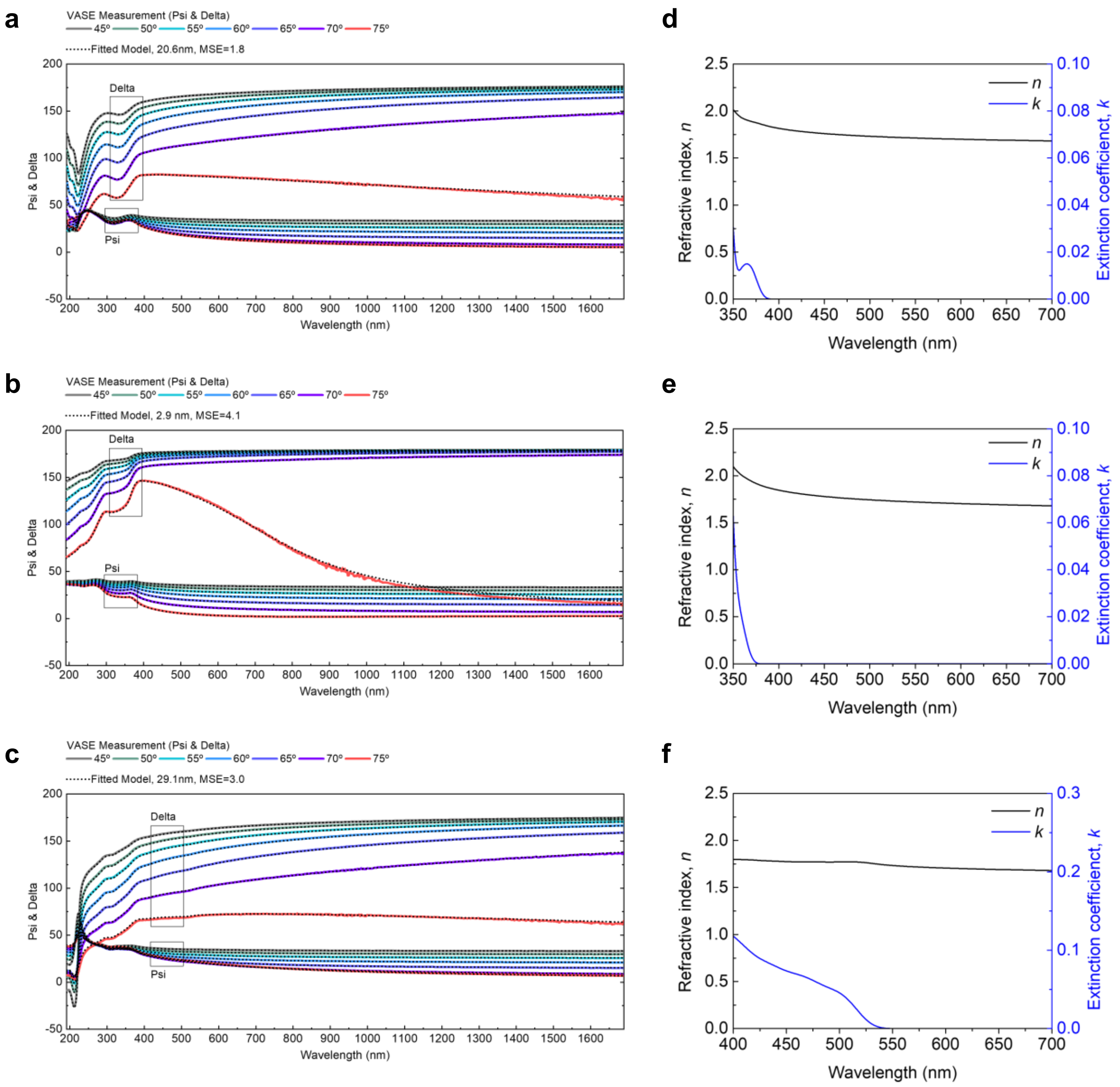

Supplementary Fig. 4 | VASE measurement of spin-coated TPBi CMM and PeNC/CMM film. a-c. Experimental psi and delta data (solid lines) obtained by VASE measurement and modeled psi and delta data (dashed lines) obtained by fitting the curves using thickness and refractive indices (n, k) as parameters. The films were fabricated using 5 mM TPBi CMM solution (**a**), 0.5 mM TPBi CMM solution (**b**), and TPBi-mixed PeNC solution (**c**). **d-f.** Obtained refractive indices (n, k) from VASE measurement and modeling. The films were fabricated using 5 mM TPBi CMM solution (**d**), 0.5 mM TPBi CMM solution (**e**), and TPBi-mixed PeNC solution (**f**).

Considering that the volume ratio of PeNC solution and CMM solution (5 mM) is 10:1 when fabricating CMM-mixed PeNC film, the amount of CMM in CMM-mixed PeNC film should be similar to the amount of a spin-coated CMM film that was fabricated using a 0.5 mM TPBi solution. The thickness of spin-coated 0.5 mM TPBi CMM solution was 2.9 nm and the thickness of TPBi mixed PeNC film was 29.1 nm Therefore, the amount of TPBi in TPBi-mixed PeNC film should be about 10 vol%.

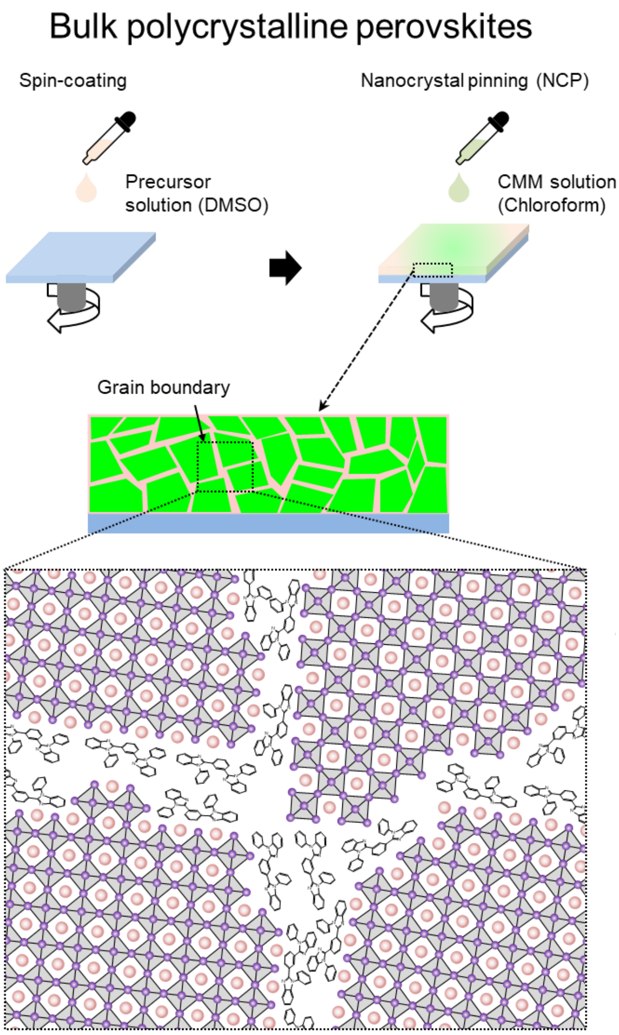

Supplementary Fig. 5 | Fabrication process of bulk polycrystalline perovskite films embedding CMMs. After dropping the precursor solution on the substrate, a spin-coating process is performed. During the spin coating process, the non-polar CMM solution is dropped, which is the nanocrystal pinning (NCP) process. The NCP process induces instantaneous nucleation and suppressed crystal growth, leading to small-sized perovskite grains. CMMs are located at the grain boundary and interact with the perovskite crystal surface.

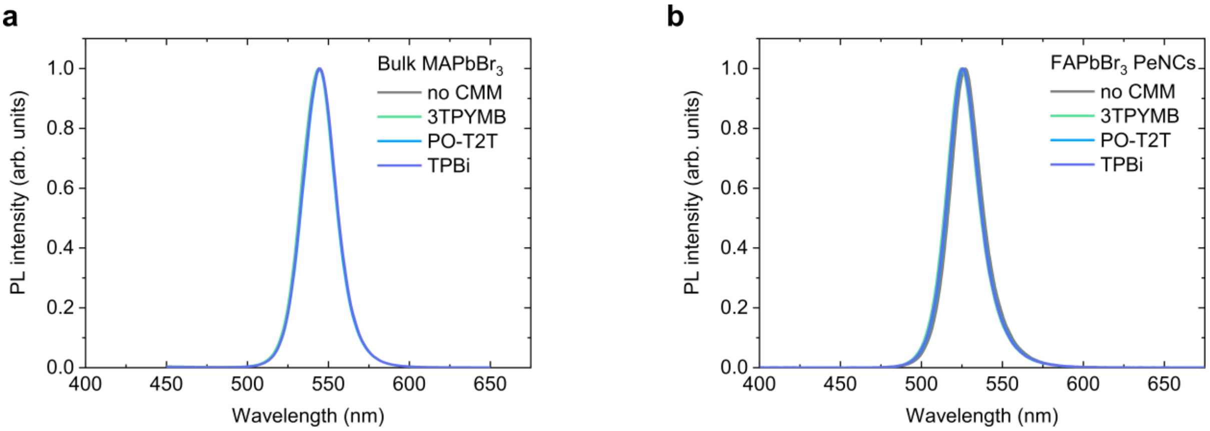

Supplementary Fig. 6 | PL spectrum of perovskite films with CMMs. a, bulk $MAPbBr_3$ with CMMs. **b**, $FAPbBr_3$ PeNC with CMMs.

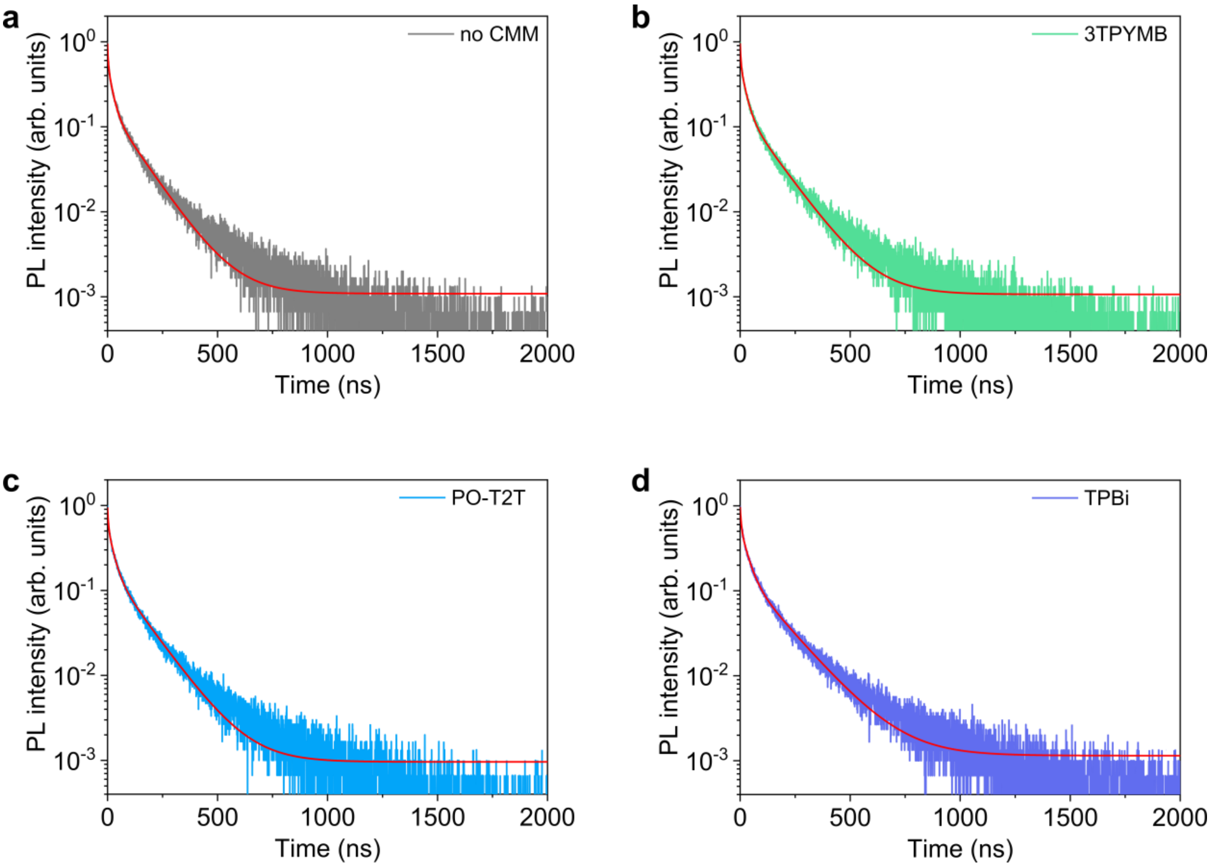

Supplementary Fig. 7 | Transient PL decay of $FAPbBr_3$ PeNC films fitted by using a tri-exponential decay function. a, no CMM, **b**, 3TPYMB, **c**, PO-T2T, and **d**, TPBi.

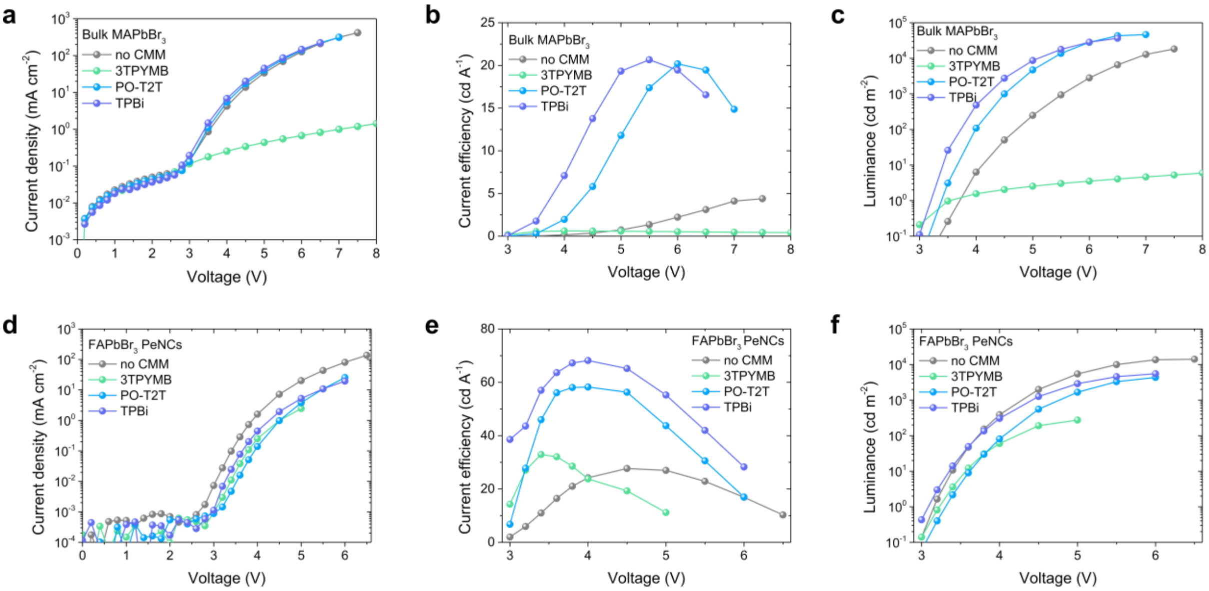

Supplementary Fig. 8 | EL characteristics of bulk $MAPbBr_3$ and $FAPbBr_3$ PeNCs embedding CMMs. a-c, Bulk $MAPbBr_3$ PeLEDs. Current density-voltage (**a**), current efficiency-voltage (**b**), and luminance-voltage (**c**). **d-f, $FAPbBr_3$ PeNC-LEDs.** Current density-voltage (**d**), current efficiency-voltage (**e**), and luminance-voltage (**f**).

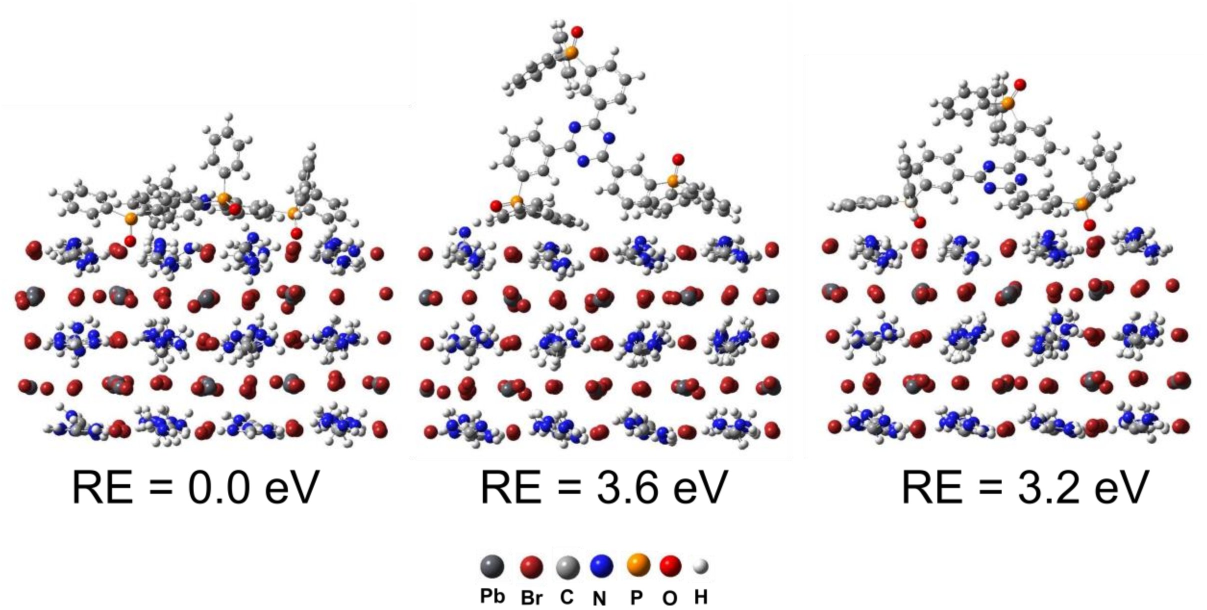

Supplementary Fig. 9 | DFT-optimized structures of PO-T2T/$FAPbBr_3$ system with different interaction orientations of PO-T2T. Relative energies (RE) compared to the most stable horizontal orientation are shown in the figure.

Horizontal orientation (left) is most stable because it maximizes the hydrogen bonding and vdW interactions between the CMM and the perovskite surface.

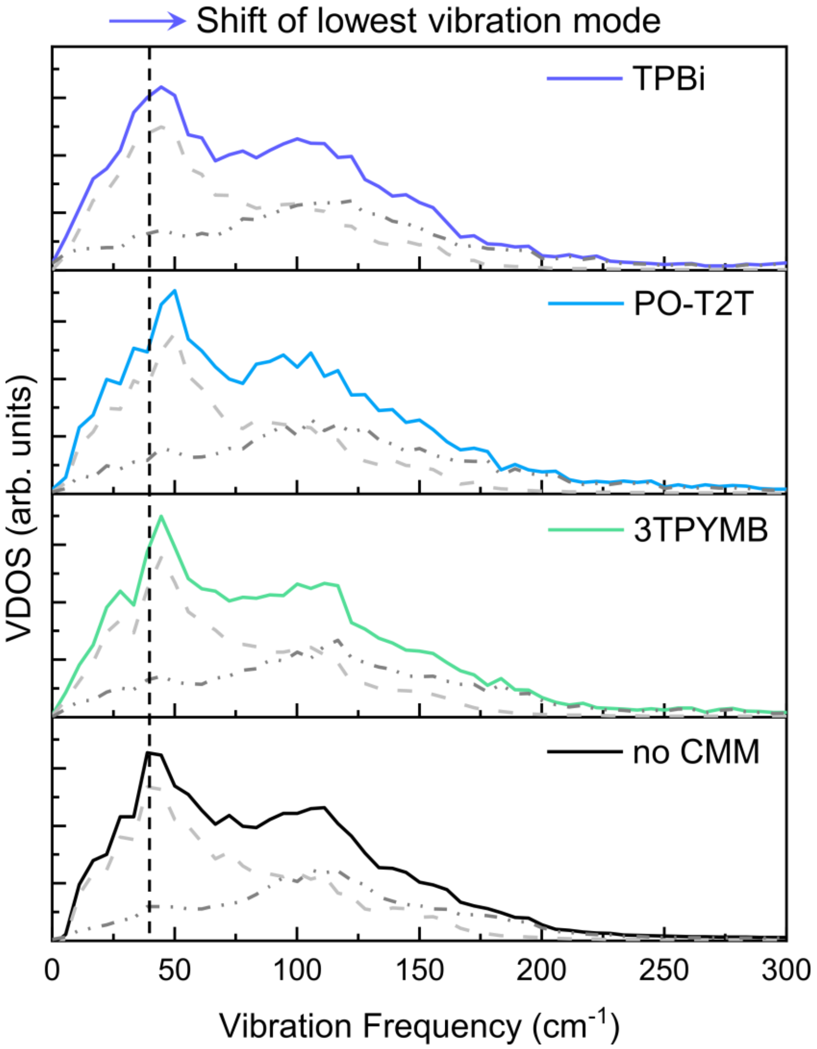

Supplementary Fig. 10 | VDOS of CMM-$FAPbBr_3$ systems. Solid lines, dashed lines, and dash-dot-dot lines indicate total, $PbBr_6$ lattice, and FA cations contributions, respectively.

All CMMs lead to a slight blue shift of the lowest vibrational mode due to the lattice-strengthening effect via hydrogen bonding and vdW interaction.

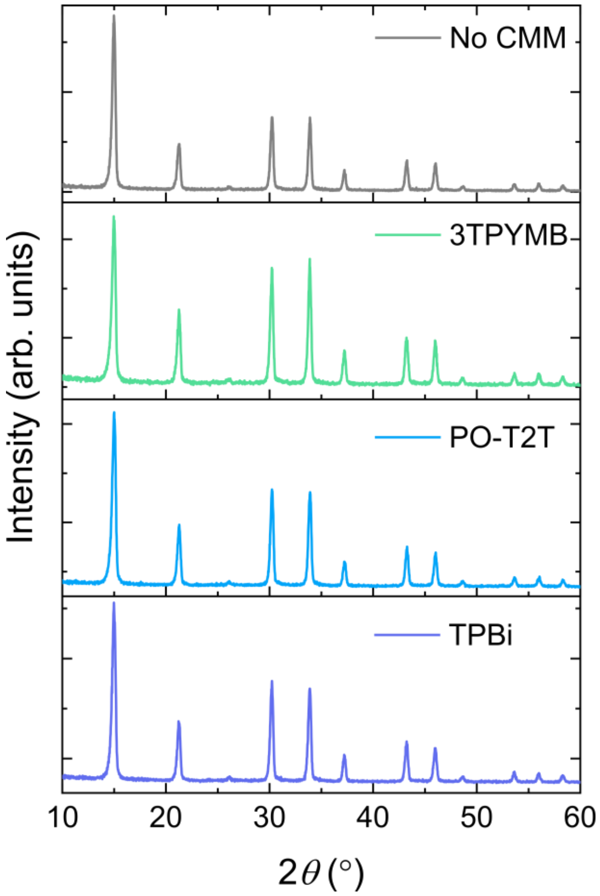

Supplementary Fig. 11 | XRD patterns of bulk $MAPbBr_3$ with CMMs. XRD patterns shows that bulk polycrystalline $MAPbBr_3$ with and without CMMs has a cubic phase. There is no peak shift upon the addition of CMMs.

XRD patterns of bulk $MAPbBr_3$ with CMMs were measured to identify the effect of CMMs on crystal structure. Bulk $MAPbBr_3$ film with no CMM showed peaks at 14.98°, 21.26°, 30.26°, 33.9°, 37.24°, 43.28°, and 46.04°, which can be assigned (100), (110), (200), (210), (211), (220) and (300) planes, respectively. These peak positions correspond to the cubic phase of $MAPbBr_3$[4]. Furthermore, there was no peak shift when CMMs were incorporated into bulk $MAPbBr_3$, indicating that CMMs do not affect the crystal structure of bulk $MAPbBr_3$.

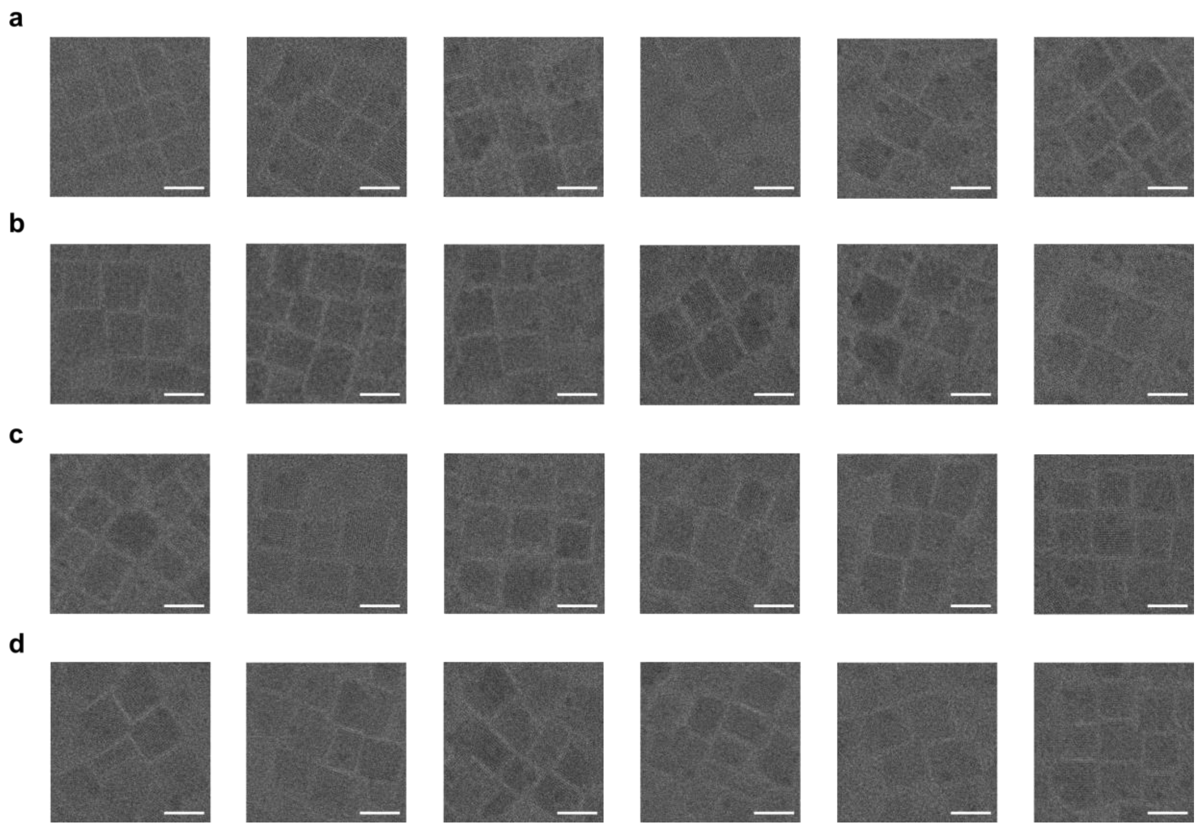

Supplementary Fig. 12 | TEM images of colloidal $FAPbBr_3$ PeNCs with CMMs. a, no CMM, **b**, 3TPYMB, **c**, PO-T2T and **d**, TPBi. Scale bar: 10 nm.

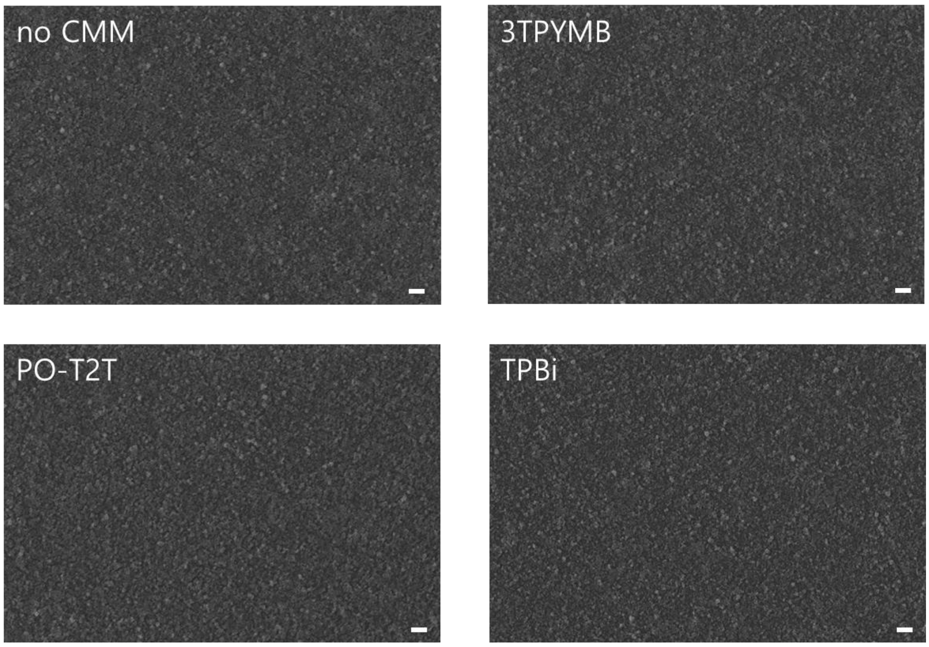

Supplementary Fig. 13 | Scanning electron microscope (SEM) images of $FAPbBr_3$ PeNC films embedding CMMs. Scale bar: 200 nm.

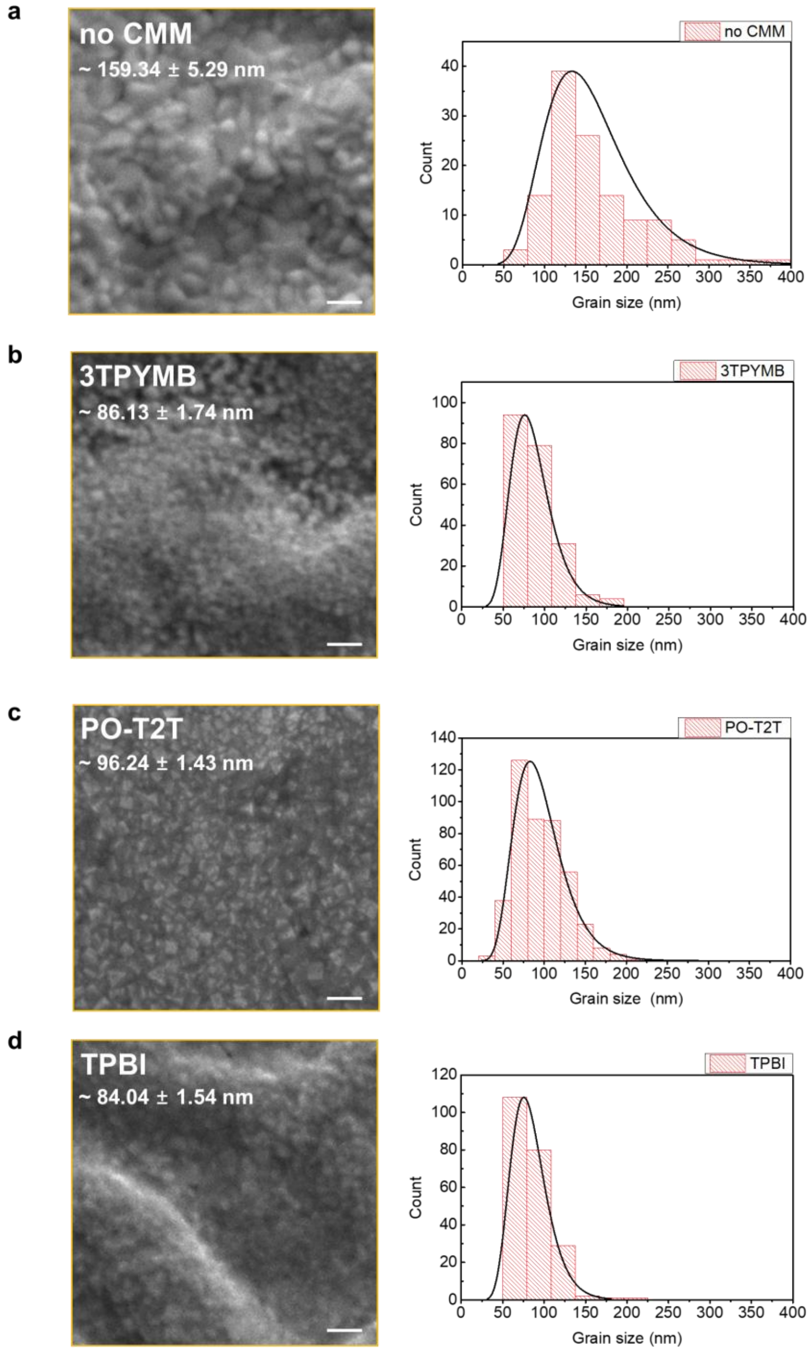

Supplementary Fig. 14 | SEM images of bulk $MAPbBr_3$ films embedding CMMs with grain size distribution. a, no CMM, **b**, 3TPYMB, **c**, PO-T2T and **d**, TPBi. Average grain size and standard error were shown. Scale bar: 200 nm.

To investigate the morphological effect of CMMs on bulk $MAPbBr_3$, we measured SEM images of CMM-embedded bulk $MAPbBr_3$ films. Bulk $MAPbBr_3$ with no CMM showed an average grain size of 159.34 ± 5.29 nm. Because CMMs are embedded into perovskite during the crystallization process (additive-based nanocrystal pinning effect)[4], the incorporation of 3TPYMB, PO-T2T, and TPBi reduced average grain size to 86.13 ± 1.74 nm, 96.24 ± 1.43 nm, and 84.04 ± 1.54 nm, respectively, which are very similar. We could not find a clear correlation between morphology and luminescent efficiency of bulk $MAPbBr_3$ upon incorporation of various CMM (Fig. 2 and Supplementary Fig. 8d-f).

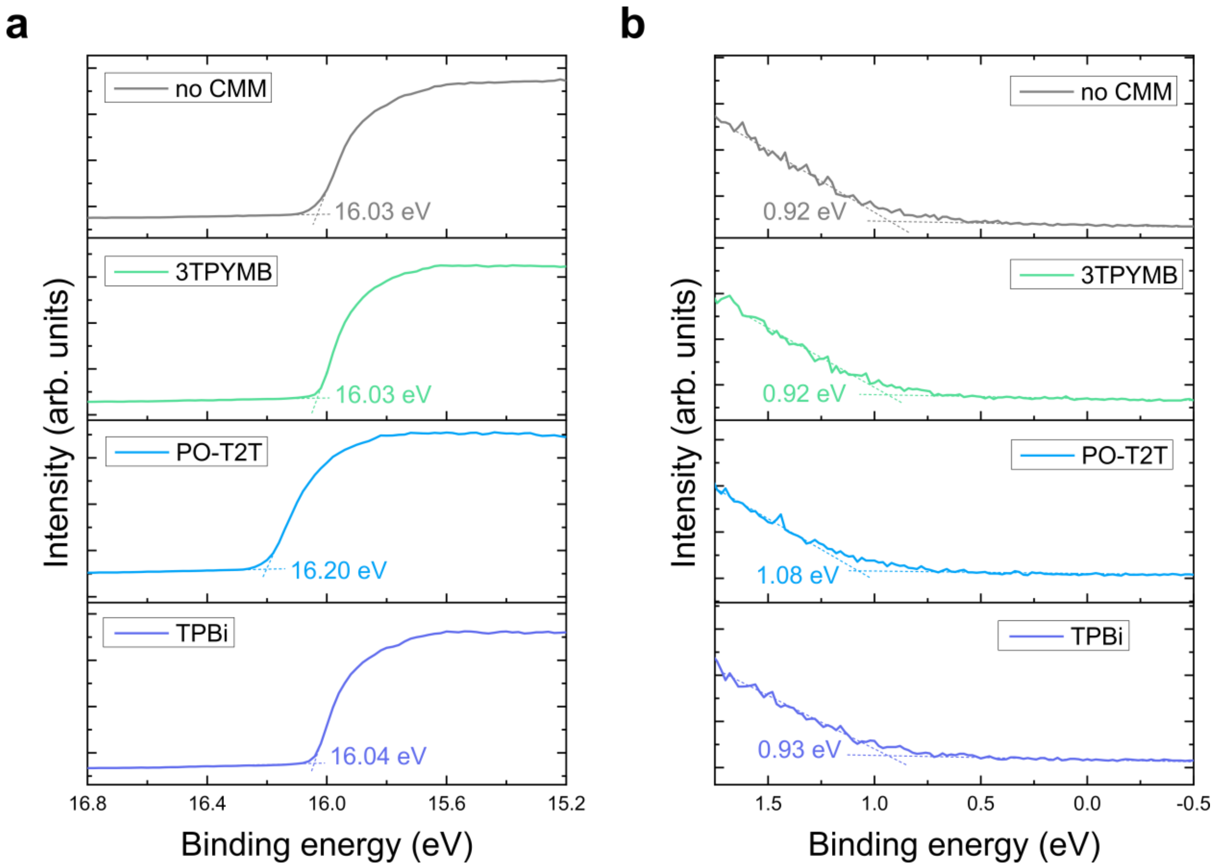

Supplementary Fig. 15 | UPS spectra of $FAPbBr_3$ PeNC films with CMMs. a, secondary cut-off. **b**, on-set. The work function of $FAPbBr_3$ PeNC with CMMs was calculated using the energies at secondary cut-off and ultraviolet radiation energy (21.2 eV). The valence band maximum level of $FAPbBr_3$ PeNC with CMMs was determined from the on-set levels and work function.

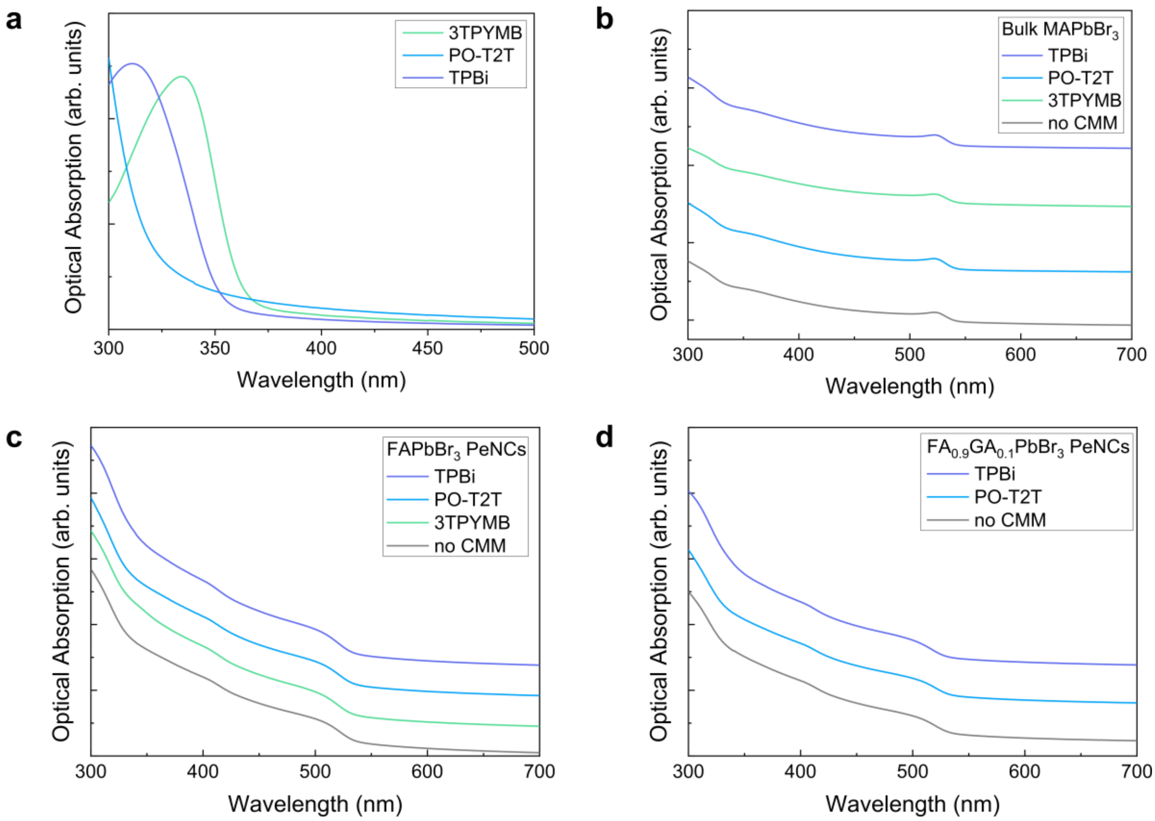

Supplementary Fig. 16 | UV-vis absorption spectra. a, CMM films, **b-d,** Perovskite films with CMMs. bulk $MAPbBr_3$ (**b**), $FAPbBr_3$ PeNCs (**c**), $FA_{0.9}GA_{0.1}PbBr_3$ PeNCs (**d**).

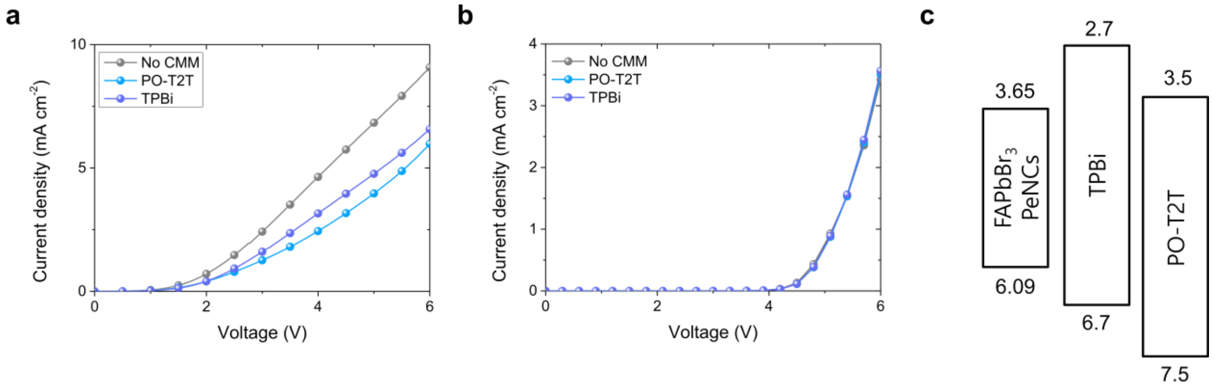

Supplementary Fig. 17 | Hole-only and electron-only device based on colloidal $FAPbBr_3$ PeNCs with CMMs. a, hole-only device, **b**, electron-only device, **c**, energy band diagram of $FAPbBr_3$ PeNCs, TPBi, and PO-T2T.

To investigate how electron-transporting CMMs affect current density in colloidal PeNC-LEDs, we compared the current density of hole-only and electron-only devices. The structure of hole-only devices was ITO (70 nm)/GraHIL (60 nm)/$FAPbBr_3$ PeNC (30 nm)/ 4,4′,4″-tris(*N*-carbazolyl)-triphenylamine (TCTA) (50 nm)/Molybdenum(VI) oxide (MoO_3) (5 nm)/Al (100 nm). The structure of the electron-only device was ITO (70 nm)/polyethyleneimine ethoxylated (PEIE) (10 nm)/$FAPbBr_3$ PeNC (30 nm)/TPBi (45 nm)/LiF (1 nm)/Al (100 nm). Although PO-T2T lowered the work function of $FAPbBr_3$ PeNCs by 0.17 eV (Fig. 4c and Supplementary Fig. 15), electron-only devices with no CMM, with TPBi and with PO-T2T showed similar current density. On the contrary, adding TPBi and PO-T2T into $FAPbBr_3$ PeNCs reduced the current density of hole-only devices. Moreover, as the HOMO level of CMMs was deepened, the current density of hole-only devices decreased more. Therefore, reduced current density in $FAPbBr_3$ PeNC-LEDs can be attributed to decreased hole current by the hole-blocking characteristics of CMMs (Supplementary Fig. 8d).

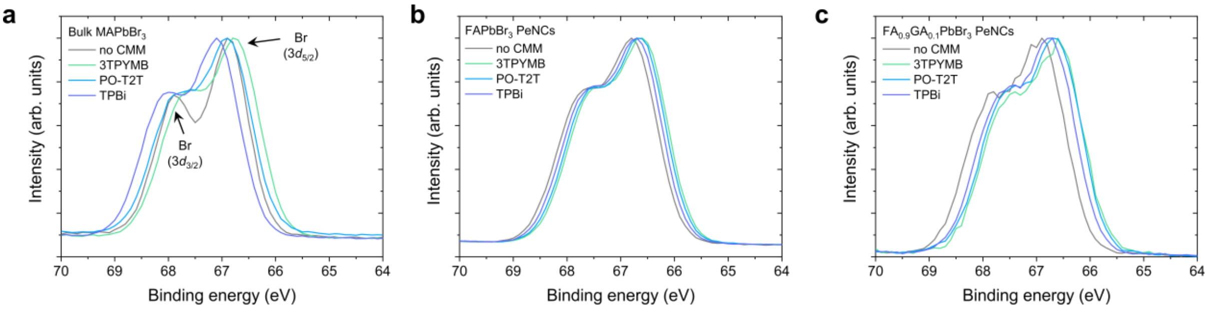

Supplementary Fig. 18 | Br 3*d* XPS spectra of perovskite films with and without CMMs. **a**, bulk $MAPbBr_3$, **b**, $FAPbBr_3$ PeNCs, **c**, $FA_{0.9}GA_{0.1}PbBr_3$ PeNCs. Additional covalent bonds were not observed.

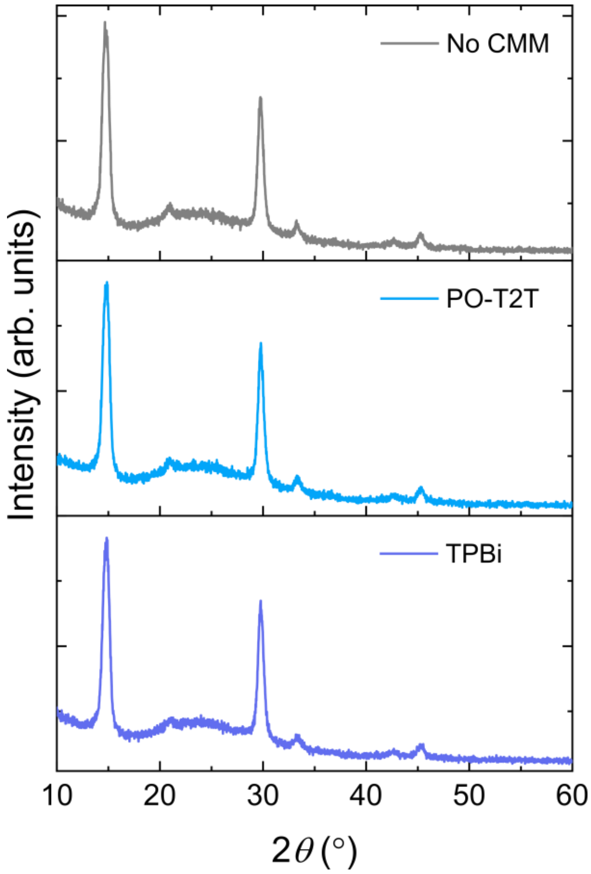

Supplementary Fig. 19 | XRD patterns of $FA_{0.9}GA_{0.1}PbBr_3$ PeNC films with CMMs. There is no peak shift upon the addition of CMMs, indicating that CMMs do not affect the crystal structure of $FA_{0.9}GA_{0.1}PbBr_3$ PeNCs.

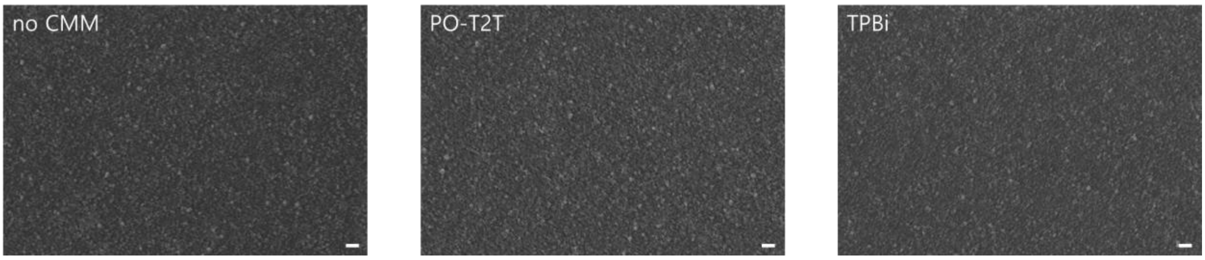

Supplementary Fig. 20 | SEM images of $FA_{0.9}GA_{0.1}PbBr_3$ PeNC films embedding CMMs. Scale bar: 200 nm.

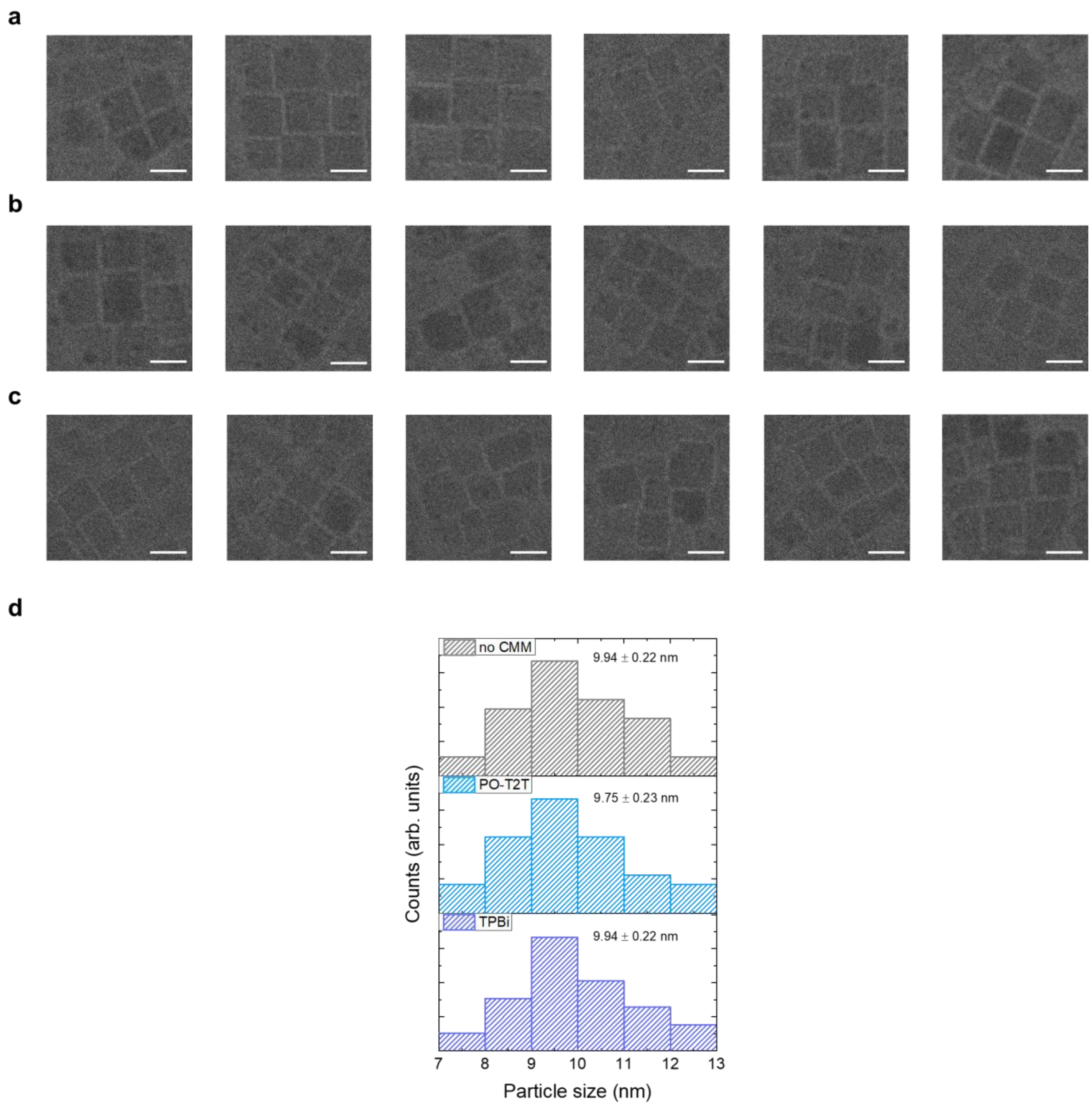

Supplementary Fig. 21 | TEM images of colloidal $FA_{0.9}GA_{0.1}PbBr_3$ PeNCs with CMMs. a, no CMM, **b**, PO-T2T, **c**, TPBi, and **d**, size distributions. Average particle size and standard error were calculated. Scale bar: 10 nm.

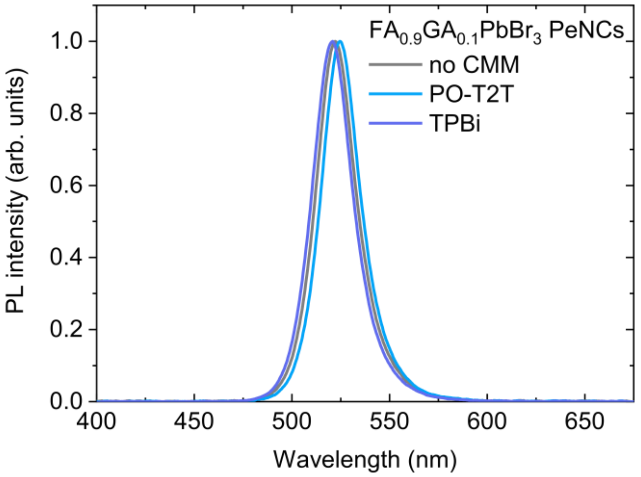

Supplementary Fig. 22 | PL spectrum of $FA_{0.9}GA_{0.1}PbBr_3$ PeNC films with CMMs. Compared to no CMM, PO-T2T showed a red-shifted PL emission, which might be attributed to the formation of metallic Pb defects. The blue-shifted PL emission by a TPBi CMM could be induced by reduced self-absorption effect due to increased interparticle spacing.

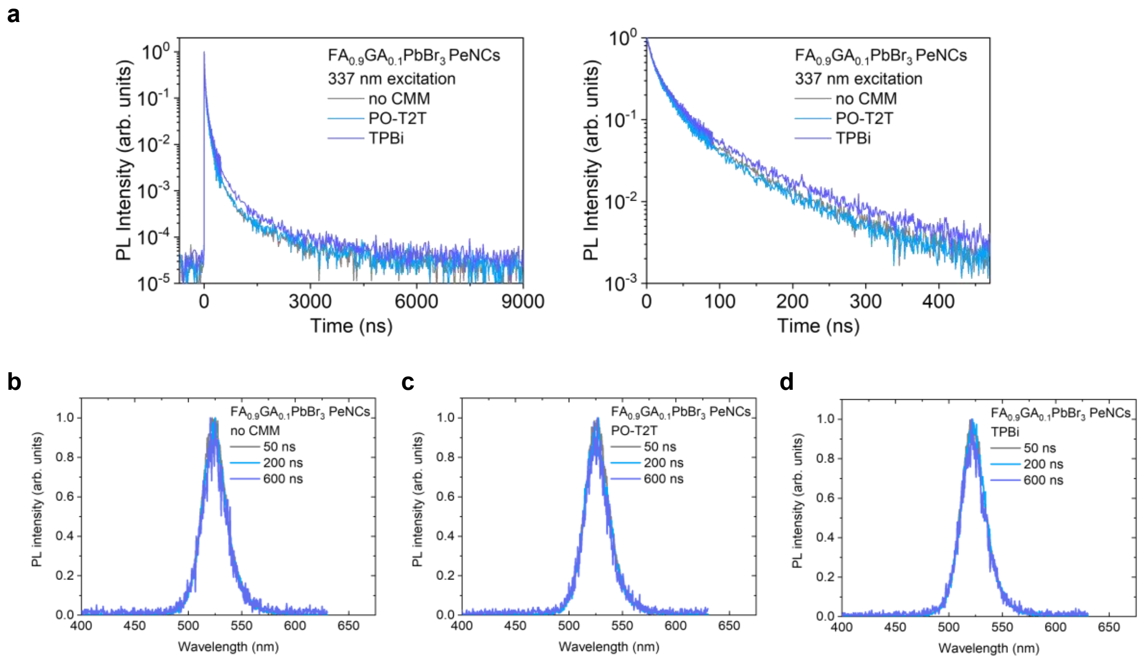

Supplementary Fig. 23 | Transient PL decay of $FA_{0.9}GA_{0.1}PbBr_3$ PeNC films with CMMs with 337 nm laser excitation. a, Transient PL decay, **b-d,** PL spectrum with varying the decay time. No CMM (**b**), PO-T2T (**c**), and TPBi (**d**).

Transient PL decay with 337 nm N_2 laser excitation which excites CMMs also shows the same trend observed from the transient PL decay with 405 nm excitation (Fig. 5e) indicating that prolonged decay lifetime of TPBi-treated PeNC didn’t originate from the energy transfer between CMM and PeNCs. In addition, an identical PL spectrum irrespective of the decay time indicated that the energy transfer had little effect on the enhanced luminescence mechanism.

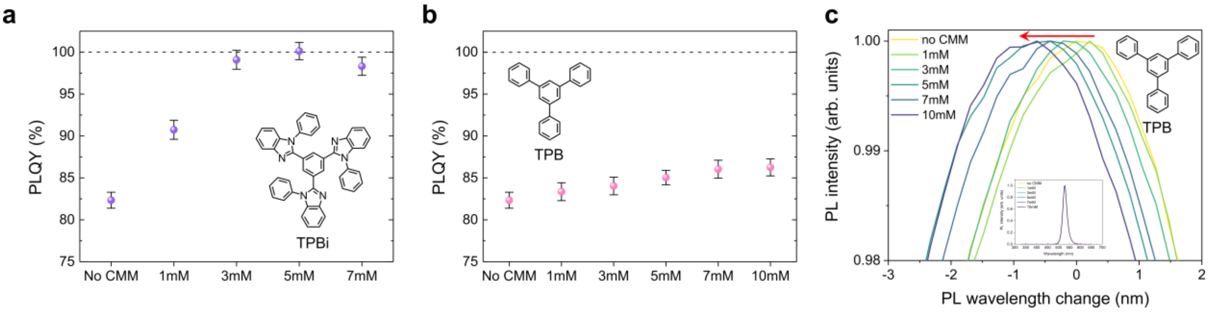

Supplementary Fig. 24 | PLQY of $FA_{0.9}GA_{0.1}PbBr_3$ PeNC films depending on the concentration of CMM solutions. a, TPBi CMM. **b-c,** Triphenylbenzene (TPB). PLQY (**b**) and PL spectrum (**c**) of $FA_{0.9}GA_{0.1}PbBr_3$ with TPB. The error bars are the standard error.

To investigate how the content of CMMs influences PLQY, we adjusted the concentration of CMM solutions. As the concentration of TPBi solution increased, the effect of lattice-strengthening increased. The PLQY was highest (near-unity) when TPBi solution was 5 mM. To clarify that the improved PLQY is due to lattice-strengthening rather than reduced self-absorption related to inter-particle spacing, triphenylbenzene (TPB), which has no functional group, was incorporated into $FA_{0.9}GA_{0.1}PbBr_3$ PeNC. As the concentration of TPB was increased, the PLQY of $FA_{0.9}GA_{0.1}PbBr_3$ increased slightly, and concurrently the PL spectrum was blue-shifted (by ~1 nm), possibly because self-absorption was reduced by increased inter-particle spacing. However, near-unity PLQY could not be achieved by only the reduced self-absorption effect. These contrasting results of TPBi and TPB highlight the importance of lattice-strengthening at the perovskite surface to increase luminescent efficiency.

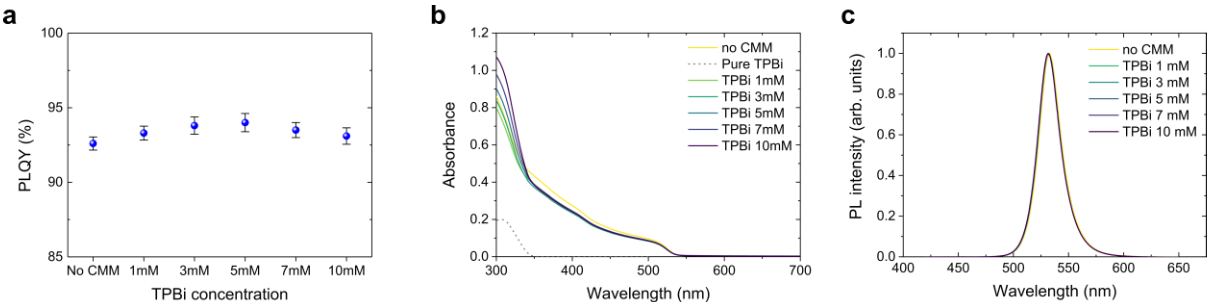

Supplementary Fig. 25 | Effect of TPBi CMM on colloidal $FA_{0.9}GA_{0.1}PbBr_3$ PeNC solution depending on the concentration of TPBi solution. **a**, PLQY. The error bars are the standard error. **b**, UV-vis absorption spectra. **c**, PL spectrum.

To confirm the presence of a lattice-strengthening effect in the colloidal PeNC solution, we measured UV-vis absorption spectra and PLQY of $FA_{0.9}GA_{0.1}PbBr_3$-TPBi mixed solutions of different TPBi concentrations. Incorporating TPBi CMM yielded a slight increase in the PLQY of colloidal $FA_{0.9}GA_{0.1}PbBr_3$ PeNC solution, but the effect was not significant because molecules and ligands in the solution phase move relatively freely and continuously detach and attach[5]. Therefore, the extent of the lattice-strengthening effect was lower in the solution than in the film. However, when spin-coating colloidal $FA_{0.9}GA_{0.1}PbBr_3$ solution containing TPBi CMM, the TPBi CMM anchors at the perovskite surface where the organic ligands are not passivating, leading to more effective lattice strengthening (Supplementary Fig. 24).

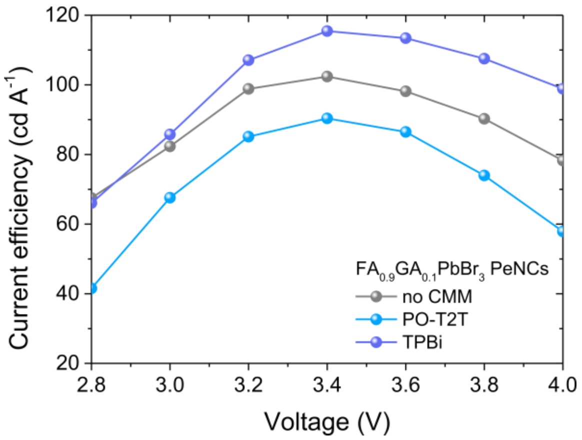

Supplementary Fig. 26 | Current efficiency curve of colloidal $FA_{0.9}GA_{0.1}PbBr_3$ PeNC devices with CMMs. The colloidal $FA_{0.9}GA_{0.1}PbBr_3$ PeNC-LED with a TPBi CMM showed an improved current efficiency of 115.4 cd A^{-1} due to the lattice-strengthening effect.

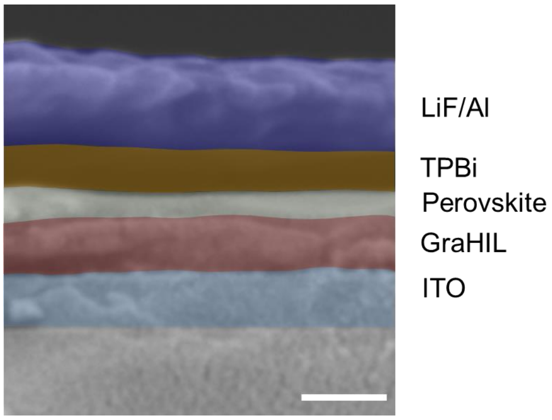

Supplementary Fig. 27 | Cross-sectional SEM image of colloidal $FA_{0.9}GA_{0.1}PbBr_3$ PeNC-LED. Scale bar: 100 nm

To ensure that the thickness of each layer in the device was correct, we measured a cross-sectional SEM image of colloidal $FA_{0.9}GA_{0.1}PbBr_3$ PeNC-LED. We confirmed that the thickness of $FA_{0.9}GA_{0.1}PbBr_3$ PeNC is 30 nm, which is consistent with the thickness measured by VASE (Supplementary Fig. 4). In addition, the thickness of the other layers was found to be consistent with the thickness we described (Supplementary Fig. 3).

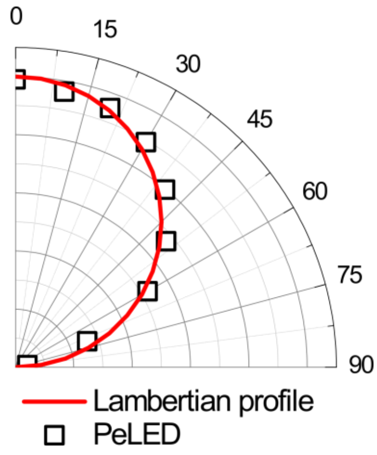

Supplementary Fig. 28 | Angle-dependent EL profile for colloidal $FA_{0.9}GA_{0.1}PbBr_3$ PeNC-LED with TPBi. EQEs of colloidal PeNC-LEDs were calculated based on angular EL distribution. Colloidal $FA_{0.9}GA_{0.1}PbBr_3$ PeNC-LED showed a Lambertian-like angle-dependent EL profile.

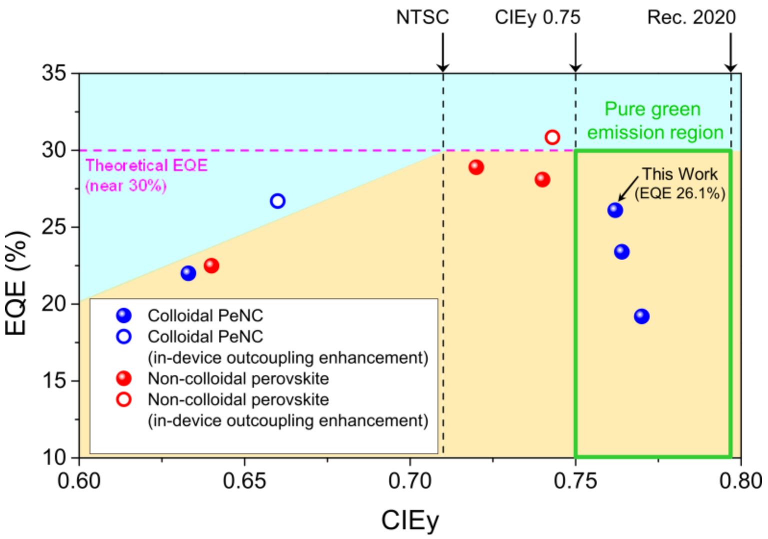

Supplementary Fig. 29 | Summary of reported high-efficiency green-emitting perovskite LEDs based on colloidal PeNC and non-colloidal perovskite (bulk perovskite). Reported EQEs of high-efficiency green-emitting PeLEDs based on colloidal PeNCs (blue circle) and non-colloidal perovskite (red circle) are summarized. EQEs of PeLEDs were categorized differently with (open circle) and without (filled circle) in-device outcoupling enhancement techniques. Detailed EL characteristics of the devices in the graph are described in Supplementary Table. 3.

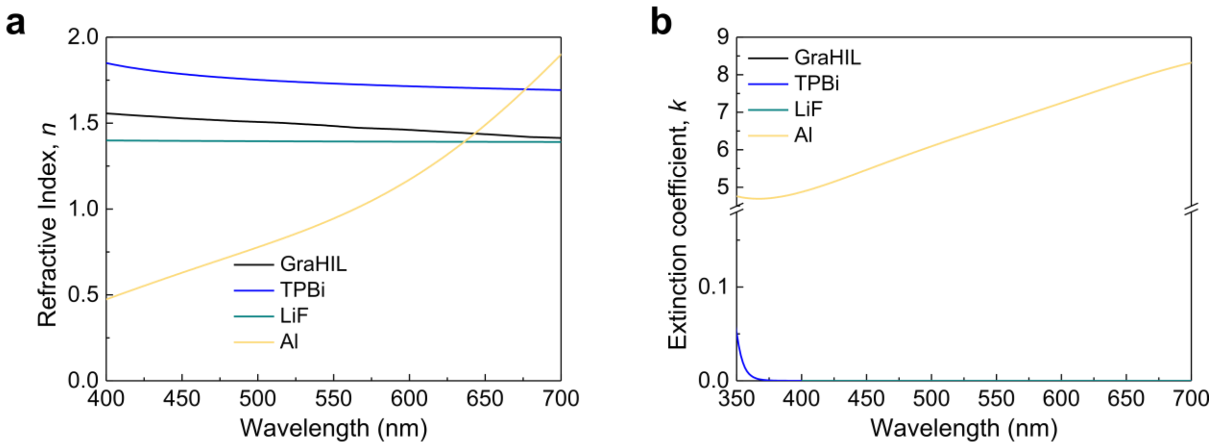

Supplementary Fig. 30 | Refractive index (*n*) and extinction coefficient (*k*) of transporting layers and electrode. a, refractive index. **b**, extinction coefficient.

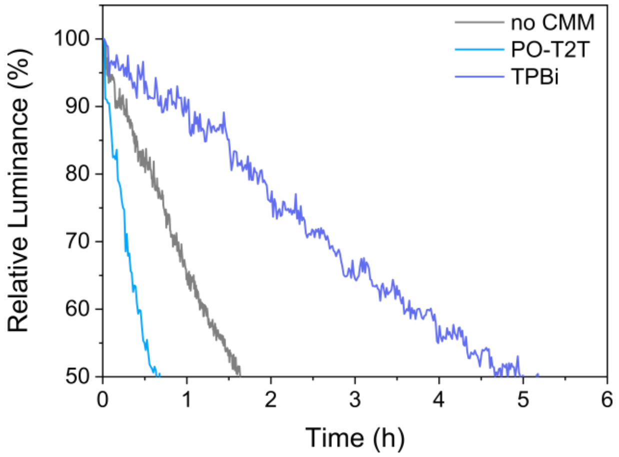

Supplementary Fig. 31 | Operational lifetime of colloidal $FA_{0.9}GA_{0.1}PbBr_3$ PeNC devices with CMMs. The operational lifetime of colloidal $FA_{0.9}GA_{0.1}PbBr_3$ PeNC-LEDs was measured under the condition with an initial luminance (L_0) of 100 cd m^{-2}.

To investigate the effect of CMM on the stability of colloidal PeNC-LEDs, we measured the device half-lifetime (LT_{50}) of colloidal $FA_{0.9}GA_{0.1}PbBr_3$ PeNC-LEDs under the condition that the initial luminance (L_0) was 100 cd m^{-2}. Compared to no CMM device (LT_{50} of 1.6h), the TPBi CMM device showed improved device lifetime (LT_{50} of 5h). On the other hand, PO-T2T exhibited reduced device stability (LT_{50} of 0.7h) because it has highly reactive characteristics with oleic acid ligand (Fig. 6). Therefore, we demonstrated that lattice-strengthening by TPBi CMM that does not induce ligand detachment not only enhances luminescent efficiency, but suppresses the degradation of perovskite.

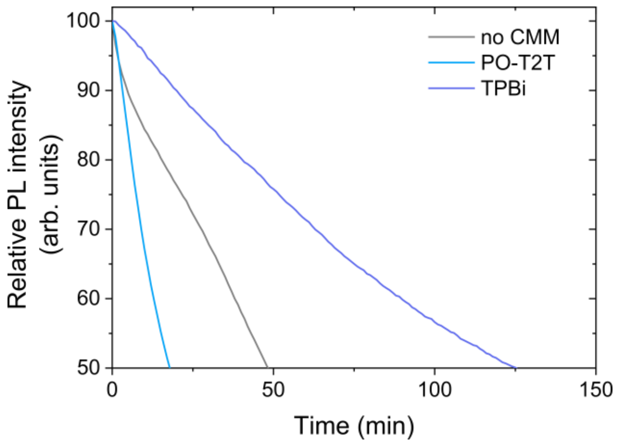

Supplementary Fig. 32 | Photostability of $FA_{0.9}GA_{0.1}PbBr_3$ PeNC films with CMMs. Encapsulated films were constantly illuminated by a 405 nm continuous-wave laser. $FA_{0.9}GA_{0.1}PbBr_3$ PeNC film with a TPBi CMM showed enhanced photostability.

The photostability of $FA_{0.9}GA_{0.1}PbBr_3$ PeNC films with CMMs was measured to verify the effect of CMMs on the stability of colloidal PeNC materials. Relative PL intensity of no CMM film decreased to 50% of its initial intensity after continuous illumination of 48 min. Compared to no CMM film, TPBi CMM films showed significantly improved photostability, retaining 50% of its initial PL intensity after 125 min. These results ensure that lattice-strengthening by a CMM enhances the stability of perovskite. However, PO-T2T CMM film showed deteriorated photostability compared to no CMM, implying that the severe ligand detachment effect rather reduces the luminescent stability of colloidal PeNCs.

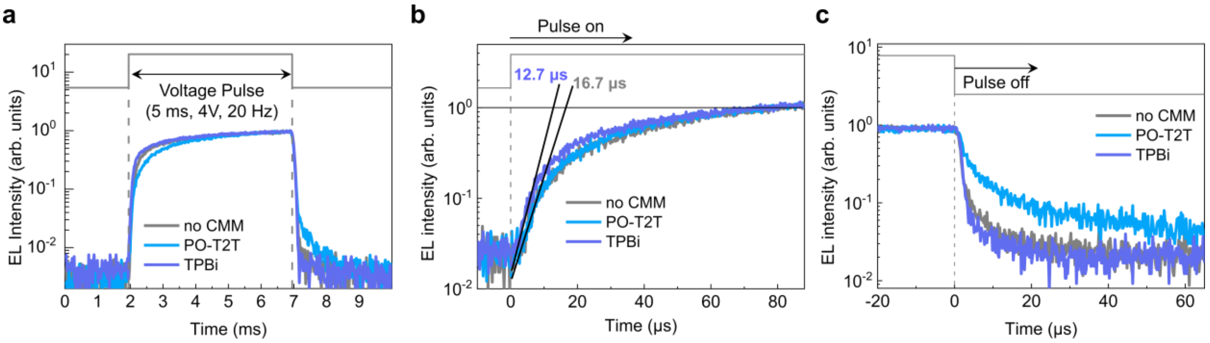

Supplementary Fig. 33 | Transient EL decay characteristics of PeNC-LEDs based on colloidal $FA_{0.9}GA_{0.1}PbBr_3$ with and without CMMs. a, Transient EL intensity of the devices during the voltage pulse of 4V and 5 milliseconds 20Hz. **b-c**, Transient EL intensity of the devices after voltage pulse on (**b**) and off (**c**).

Transient EL decay characteristics were measured to study the effect of CMMs on the EL response of colloidal $FA_{0.9}GA_{0.1}PbBr_3$ PeNC-LEDs. Due to defects generated by ligand detachment induced by PO-T2T, colloidal $FA_{0.9}GA_{0.1}PbBr_3$ PeNC-LED incorporating PO-T2T showed slower rising and falling times of the turn-on and turn-off EL response.

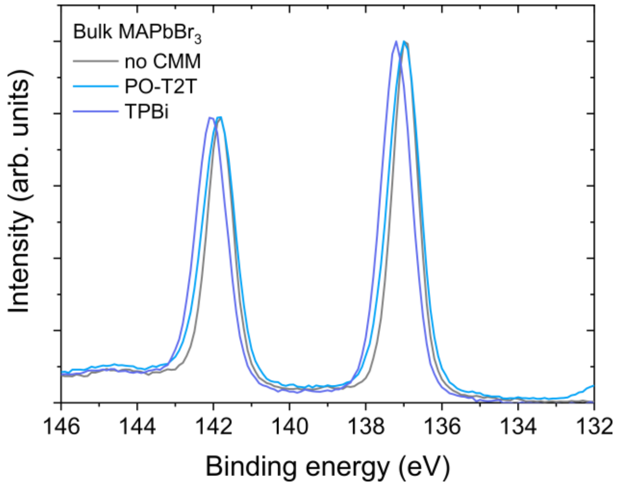

Supplementary Fig. 34 | Pb 4*f* XPS spectra of Bulk $MAPbBr_3$. Two peaks at the binding energy of ~142 eV and ~137 eV could be assigned to Pb 4$f_{7/2}$ and Pb 4$f_{5/2}$ levels. There is no formation of metallic Pb.

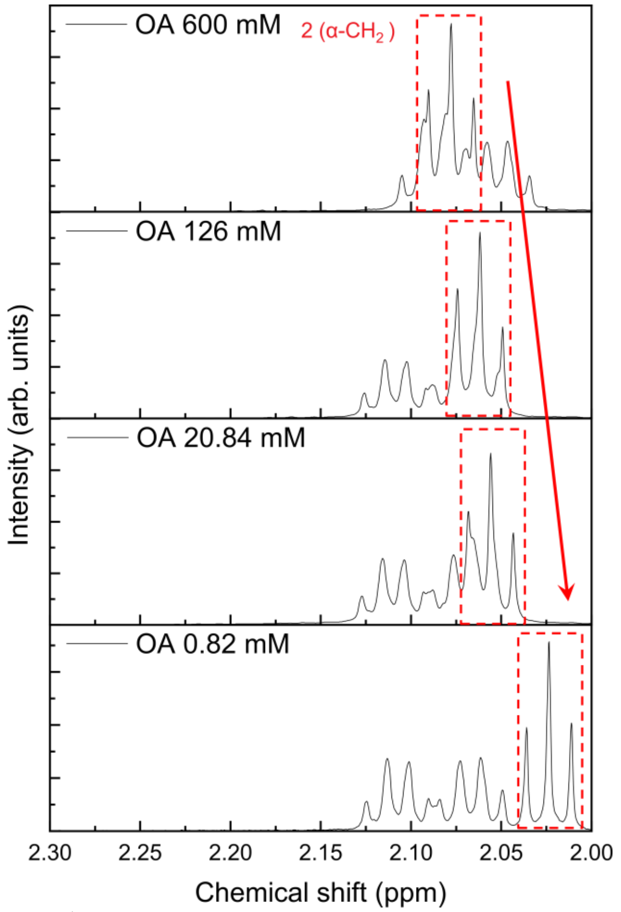

Supplementary Fig. 35 | ^{1}H-NMR spectra of oleic acid dissolved in benzene-d6 with decreasing concentration. With decreasing the concentration of oleic acid in the solvent, the peaks for α-CH_2 in oleic acid showed up-field shift due to their molecular interaction. 0.82 mM concentration of oleic acid was used to analyze the interaction between ligands and CMMs in the main text.

To assign the peaks for α-CH_2 in OA at 0.82 mM, we measured ^{1}H-NMR spectra as the concentration of OA increased. At 0.6-M concentration, the peaks at ~2.07 ppm could be assigned to α-CH_2 in OA[6,7]. Therefore, at 0.82-mM, the peaks at ~2.025 ppm could be accurately assigned to α-CH_2 in OA.

Supplementary Table. 1 | Photophysical properties of $FAPbBr_3$ PeNCs with CMMs. Average PL decay lifetime (τ_{PL}) was obtained from transient PL decay curves fitted by using a tri-exponential decay function. Radiative decay rates (k_r) and nonradiative decay rates (k_{nr}) were calculated using the values of PLQY and τ_{PL} based on the following relations. PLQY = k_r / (k_r + k_{nr}). k_r + k_{nr} = 1/τ_{PL}.

**Condition**	A_1	τ_1 (ns)	A_2	τ_2 (ns)	A_3	τ_3 (ns)	τ_{PL} (ns)	PLQY (%)	k_r ($10^6 s^{-1}$)	k_{nr} ($10^6 s^{-1}$)
no CMM	0.37593	1.52461	0.40463	13.68738	0.16871	79.07663	20.5	79.5	38.7805	10
3TPYMB	0.3899	1.90767	0.40711	15.52381	0.16344	83.87618	21.6	83.9	38.8426	7.45370
PO-T2T	0.35165	2.00532	0.41589	16.67015	0.18268	83.81514	24.2	87.2	36.0331	5.28926
TPBi	0.38975	2.84804	0.41396	21.04269	0.16515	101.2882	27.4	95.2	34.7445	1.75182

Supplementary Table. 2 | Summary of evolved performance of state-of-the-art green-emitting colloidal PeNC-LEDs without outcoupling enhancement technique and their EL emission characteristics (CIE 1931 color coordinate, EL peak, FWHM, Max. EQE, and device lifetime).

Publication date	CIE 1931 color coordinate	EL peak (nm)	FWHM (nm)	Max. EQE (%)	Device lifetime (LT_{50})	Ref.
2015/07	(0.061, 0.785)[a]	516	23	0.12	-	8
2015/12	(0.14, 0.77)	524	24	1.1	-	9
2016/05	(0.066, 0.764)[a]	525	24	1.06	10 day (L_0= unknown)	10
2016/08	(0.085, 0.770)[a]	515	19	3.0	-	11
2016/11	(0.05, 0.71)	512	20	6.27	-	12
2017/08	(0.168, 0.773)	529	22.8	3.04	-	13
2018/03	(0.20, 0.76)	532	25	13.4	800 s (L_0=105 cd m^{-2})	14
2018/09	(0.0613, 0.732)	519	18	6.04	-	15
2018/10	(0.174, 0.78)	532	22	3.53	32 min (L_0=14.2 cd m^{-2})	16
2018/10	(0.09, 0.71)	518	18	16.48	136 min (J= 0.6 mA cm^{-2})	17
2019/06	(0.172,0.765)	528	24	2.96	37 h[b]	18
2020/07	(0.032, 0.633)[a]	505	17.3[a]	22	60 min (L_0 =1200 cd m^{-2})	19
2020/08	(0.074,0.707)	516	20	18.7	15.8 h[b]	20
2021/01	(0.196, 0.764)	531	20.7	23.4	132 min (L_0 =100 cd m^{-2})	21
2021/06	(0.19,0.77)	532	21	19.2	20 min (L_0 =100 cd m^{-2})	22
2022/05	(0.187,0.767)	530	20	23.3	81 min (L_0 =100 cd m^{-2})	23

2023/09	(0.097, 0.781)[a]	518	17.8[a]	23.45	77 s (L_0 =220 cd m^{-2})	24
2023/10	(0.092, 0.766)[a]	517	16.1	24.13	54 min (L_0 =10,000 cd m^{-2})	25
-	(0.199, 0.762)	531	20.7	26.1	5h (L_0 =100 cd m^{-2})	This work

[a]Estimated value obtained from digitized data points in reported EL spectrum images
[b]Extrapolated to 100 cd m^{-2} using acceleration factor (n), $L_0^n T_{50}$ = constant

We demonstrated the high EQE of 26.1% in green-emitting colloidal PeNC-LEDs without relying on outcoupling enhancement techniques. Moreover, a lattice-strengthening CMM without ligand detachment effect enabled improvement in the real-time operational lifetime of PeNC-LEDs up to LT_{50} of 5h, comparable to real-time lifetimes of state-of-the-art PeNC-LEDs. However, the overall operational lifetime of state-of-the-art PeNC-LEDs is far behind compared to that of bulk polycrystalline PeLEDs[26]. This gap stands out the necessity to further develop properties of colloidal PeNCs by designing additive molecules and strongly surface-binding ligands that can ameliorate the stability of PeNCs. Our comprehensive understanding of the dynamic disorder of perovskite lattice and dynamic binding characteristics of the ligands in colloidal PeNCs would be a stepping stone to developing future strategies for improving the luminescent efficiency and operational lifetime of PeNC-LEDs.

Supplementary Table. 3 | Summary of high-efficiency green-emitting PeLEDs based on colloidal PeNC and non-colloidal perovskite (bulk perovskite).

Publication date	Type	Outcoupling enhancement	EL peak (nm)	CIE 1931 color coordinate	Max. EQE (%)	Ref.
2020/07	Colloidal PeNC	No	505	(0.032,0.633)[a]	22	19
2021/01	Colloidal PeNC	No	531	(0.196, 0.764)	23.4	21
2021/05	Bulk perovskite	No	508	(0.049, 0.64)	22.49	27
2021/06	Colloidal PeNC	No	532	(0.19, 0.77)	19.2	22
2021/10	Bulk perovskite	No	514	(0.10, 0.74)	28.1	28
2022/11	Bulk perovskite	No	540	(0.26, 0.72)	28.9	26
2023/01	Colloidal PeNC	Yes	514	(0.05, 0.66)	26.7	29
2023/05	Bulk perovskite	Yes	524	(0.181, 0.743)	30.84	30
-	Colloidal PeNC	No	531	(0.199, 0.762)	26.1	This work

[a]Estimated value obtained from digitized data points in reported EL spectrum images

Supplementary References

1. Park, Y. S. *et al.* Exciplex-forming Co-host for organic light-emitting diodes with ultimate efficiency. *Adv. Funct. Mater.* **23**, 4914–4920 (2013).

2. Nayak, P. K., Patankar, M. P., Narasimhan, K. L. & Periasamy, N. Excited state complex and electroluminescence in TPD-based single layer device. *J. Lumin.* **130**, 1174–1178 (2010).

3. Kalinowski, J., Giro, G., Cocchi, M., Fattori, V. & Di Marco, P. Unusual disparity in electroluminescence and photoluminescence spectra of vacuum-evaporated films of 1,1-bis ((di-4-tolylamino) phenyl) cyclohexane. *Appl. Phys. Lett.* **76**, 2352–2354 (2000).

4. Cho, H. *et al.* Overcoming the electroluminescence efficiency limitations of perovskite light-emitting diodes. *Science* **350**, 1222–1225 (2015).

5. De Roo, J. *et al.* Highly dynamic ligand binding and light absorption coefficient of cesium lead bromide perovskite nanocrystals. *ACS Nano* **10**, 2071–2081 (2016).

6. Almeida, G. *et al.* Role of acid-base equilibria in the size, shape, and phase control of cesium lead bromide nanocrystals. *ACS Nano* **12**, 1704–1711 (2018).

7. Almeida, G. *et al.* The phosphine oxide route toward lead halide perovskite nanocrystals. *J. Am. Chem. Soc.* **140**, 14878–14886 (2018).

8. Song, J. *et al.* Quantum dot light-emitting diodes based on inorganic perovskite cesium lead halides ($CsPbX_3$). *Adv. Mater.* **27**, 7162–7167 (2015).

9. Huang, H. *et al.* Emulsion synthesis of size-tunable $CH_3NH_3PbBr_3$ quantum dots: an alternative route toward efficient light-emitting diodes. *ACS Appl. Mater. Interfaces* **7**, 28128–28133 (2015).

10. Deng, W. *et al.* Organometal halide perovskite quantum dot light-emitting diodes. *Adv. Funct. Mater.* **26**, 4797–4802 (2016).

11. Pan, J. *et al.* Highly efficient perovskite-quantum-dot light-emitting diodes by surface engineering. *Adv. Mater.* **28**, 8718–8725 (2016).

12. Li, J. *et al.* 50-Fold EQE improvement up to 6.27% of solution-processed all-inorganic perovskite $CsPbBr_3$ QLEDs via surface ligand density control. *Adv. Mater.* **29**,

1603885 (2017).

13. Kumar, S. *et al.* Ultrapure green light-emitting diodes using two-dimensional formamidinium perovskites: achieving recommendation 2020 color coordinates. *Nano Lett.* **17**, 5277–5284 (2017).

14. Chin, X. Y. *et al.* Self-assembled hierarchical nanostructured perovskites enable highly efficient LEDs: via an energy cascade. *Energy Environ. Sci.* **11**, 1770–1778 (2018).

15. Di Stasio, F. *et al.* High-efficiency light-emitting diodes based on formamidinium lead bromide nanocrystals and solution processed transport layers. *Chem. Mater.* **30**, 6231–6235 (2018).

16. Fang, H. *et al.* Few-layer formamidinium lead bromide nanoplatelets for ultrapure-green and high-efficiency light-emitting diodes. *Nano Res.* **12**, 171–176 (2019).

17. Song, J. *et al.* Organic–inorganic hybrid passivation enables perovskite QLEDs with an EQE of 16.48%. *Adv. Mater.* **30**, 1805409 (2018).

18. Kumar, S. *et al.* Efficient perovskite nanocrystal light-emitting diodes using a benzimidazole-substituted anthracene derivative as the electron transport material. *J. Mater. Chem. C* **7**, 8938–8945 (2019).

19. Dong, Y. *et al.* Bipolar-shell resurfacing for blue LEDs based on strongly confined perovskite quantum dots. *Nat. Nanotechnol.* **15**, 668–674 (2020).

20. Xu, L. *et al.* A bilateral interfacial passivation strategy promoting efficiency and stability of perovskite quantum dot light-emitting diodes. *Nat. Commun.* **11**, 3902 (2020).

21. Kim, Y.-H. *et al.* Comprehensive defect suppression in perovskite nanocrystals for high-efficiency light-emitting diodes. *Nat. Photonics* **15**, 148–155 (2021).

22. Zhao, H. *et al.* High-brightness perovskite light-emitting diodes based on $FAPbBr_3$ nanocrystals with rationally designed aromatic ligands. *ACS Energy Lett.* **6**, 2395–2403 (2021).

23. Kim, Y.-H. *et al.* Exploiting the full advantages of colloidal perovskite nanocrystals for large-area efficient light-emitting diodes. *Nat. Nanotechnol.* **17**, 590–597 (2022).

24. Mei, X. *et al.* In situ ligand compensation of perovskite quantum dots for efficient light-emitting diodes. *ACS Energy Lett.* **8**, 4386–4396 (2023).

25. Zhang, X. *et al.* Stable perovskite quantum dots light-emitting diodes with efficiency exceeding 24%. *Adv. Sci.* **10**, 1–7 (2023).

26. Kim, J. S. *et al.* Ultra-bright, efficient and stable perovskite light-emitting diodes. *Nature* **611**, 688–694 (2022).

27. Chu, Z. *et al.* Perovskite light-emitting diodes with external quantum efficiency exceeding 22% via small-molecule passivation. *Adv. Mater.* **33**, 1–9 (2021).

28. Liu, Z. *et al.* Perovskite light-emitting diodes with EQE exceeding 28% through a synergetic dual-additive strategy for defect passivation and nanostructure regulation. *Adv. Mater.* **33**, 1–9 (2021).

29. Wan, Q. *et al.* Ultrathin light-emitting diodes with external efficiency over 26% based on resurfaced perovskite nanocrystals. *ACS Energy Lett.* **8**, 927–934 (2023).

30. Bai, W. *et al.* Perovskite light-emitting diodes with an external quantum efficiency exceeding 30%. *Adv. Mater.* **35**, 1–8 (2023).
